# Recreation in Relation to the Health of the People

**Published:** 1891-06-27

**Authors:** 


### RECREATION IN RELATION TO THE HEALTH OF THE PEOPLE.

### IV.—RECREATION FOR THE MIND.

POPULAR lectures do not, perhaps, appeal to so large a proportion of the uncultured classes as do popular concerts; but there are few human beings, after all, in whose minds cannot be roused feelings of wonder and curiosity as to the many things which lie around them, yet are out of their ken; and if the subjects are judiciously chosen, and the lectures themselves clearly and brightly delivered, they do not fail to attract keen interest. We choose at random a few of the titles of the excellent series of penny lectures delivered, season by season, at the Victoria Hall: "How Soap is Made," "Pygmies," "The Phonograph," "Science in the Saucepan," "Spinning Tops," "King Frost." It can readily be understood that simple and chatty lectures on such subjects are appreciated, even by those who have never troubled themselves to think; and when once an appetite for thinking and learning is acquired, the way will have been paved, not only for classes, but for lectures of a higher type.

Of this latter class are the lectures which form so important a feature in the work of Toynbee Hall and Oxford House. The Oxford House lectures are given, not only at the Oxford House, but at such London working men's clubs as choose to apply for them, such subjects being taken as "The Laws We Live Under," "Father Damian," "Forms of Government," "Animal Intelligence," etc., etc. Another series of lectures, on subjects connected directly or indirectly with religion, is given on Sunday afternoons; and we may also mention that, at Toynbee Hall, conferences, or "Smoking Debates," are frequently held on such subjects as "Work and Wages of Women," "Water Rates," "Peasant Proprietary," etc.

That popular lectures, like popular concerts, do pave the way for something higher is shown by the experience of the managers of the Victoria Hall. "We found," say they, "that our lectures had awakened an interest in Science and Nature greater than could be satisfied by mere isolated lectures, and we were asked for some systematic instruction to which they might act as feeders." To satisfy these desires the Morley Memorial College was opened; and now various kinds of classes are held there, both for men and women, gymnasia and recreation and reading-rooms also being provided. Both here and at the People's Palace, the excellence and variety of whose classes are well known, recreation and education (or, shall we say, the two kinds of recreation?) are made, as far as possible, to go hand-in-hand, only those who attend at least one of the classes being allowed to share in the social and recreative advantages which are here to be obtained.

We do not dwell at any length on classes such as these, because in so doing we might seem to be trenching upon other matters than that with which we are now concerned. The moment such classes are attended—as many of them, of course, are—with the object of obtaining a better knowledge of a certain trade, or of gaining some accomplishment which will better enable the student to earn a livelihood (such, for instance, as colloquial French, which a certain cabby has been observed to be most intent on acquiring), they cease to come under the head of recreation; and it is by no means easy to draw the line between the two classes of students—those who labour with an eye to bread-and-butter, and those who learn for the love of learning, and, in the best sense of the word, recreating themselves.

The Recreative Evening Schools Association, perhaps, supplies us with a better instance of work undertaken simply for its educational and recreative advantages; and very useful work this Association is doing. It aims at introducing, both in voluntary and in Board schools, continuation classes of various kinds, vocal and instrumental music, wood-carving, clay-modelling, musical drill, cooking, domestic economy, and many other things, being taught; while such subjects as history, geography, and science are made attractive by means of the lantern, and free-and-easy talks rather than lectures.

We must now turn to music, and see how in this department also the plan of beginning at the bottom proves successful. It has already been shown that the taste of the people can be raised, so that a concert which would once have been far above their heads is now received with eager attention and delight; it must now be chronicled that the love for good music which has thus been engendered is not satisfied with only hearing it, but cries out to be allowed a share in its production. The Popular Musical Union reports that its musical performances become every year less and less concerts of the usual description, but rather oratorios and works performed by the choir and orchestra trained in their classes at—Kensington or Westminster, perhaps? Nothing of the kind! Bermondsey, Clerkenwell, and Whitechapel, if you please. And the *Times* reporter, who was present at one of these performances, declared that "the choir had excellent tone, and in the matter of rhythmic precision might put many a West-end choir to the blush."

We do not see that much is done by those who endeavour to provide recreation for the people in the way of satisfying that love of acting which is to be found among the poor, quite as much as, if not more than, among the rich. And as regards the love of taking a personal share in dramatic performances, this is probably just as well, for there are many difficulties connected with the subject, and wise and constant supervision must always be necessary—more, probably, than could generally be given. Oxford House, however, has a successful Dramatic Society, which gives performances at the quarterly "Evening Parties," and in other parts of London; and we may add that where it is desired to find some light and attractive amusement for a class of girls alone, or of lads alone, the working up of a simple play will excite great interest, and can be managed with comparatively little trouble.

On the whole, however, it seems best, in view of the temptations and unsettlingness (if we may coin a word) of "play-acting," especially with regard to women and girls, to avoid spreading a love for it, and to give opportunities for seeing good acting, rather than for taking a share in it. Does this sound rather heartless—caring for our own *protégés* at the expense of those who minister to our pleasures? We do not mean it so. Acting, like horsebreaking, is a dangerous profession—there can be no doubt of that, to our thinking; and we do not want all people to run a risk of breaking their necks, because some people must. That our instinctive love of acting will never die out, and that there will, therefore, always be actors and actresses, is all but certain; but we need not swell their ranks more than is necessary, and meanwhile, we can do much to help them (just as we have already helped music-hall singers) by encouraging the production of good and wholesome plays, and by raising the taste of their audiences.

Good work is already being done in this way, we do not doubt, by the Oxford House Dramatic Society already mentioned, and by the Recreation Society, St. Peter's, London Docks;* and it is much to be hoped, as the Victoria Hall Committee remarks, that the vexatious restrictions of the law as it at present stands with regard to this matter may ere long be removed, and the public more efficiently catered for by those who have its best interests at heart than is at present possible.

* We do not of course profess to give in these articles anything like a complete list of the various agencies at work among the people with a view to their recreation. Such a list, though interesting, would be very lengthy, as well as difficult to compile; here we can do no more than mention such as seem best to illustrate our arguments.

